# Association between perceived insufficient sleep, frequent mental distress, obesity and chronic diseases among US adults, 2009 behavioral risk factor surveillance system

**DOI:** 10.1186/1471-2458-13-84

**Published:** 2013-01-29

**Authors:** Yong Liu, Janet B Croft, Anne G Wheaton, Geraldine S Perry, Daniel P Chapman, Tara W Strine, Lela R McKnight-Eily, Letitia Presley-Cantrell

**Affiliations:** 1 Division of Population Health, National Center for Chronic Disease Prevention and Health Promotion, Centers for Disease Control and Prevention (CDC), 4770 Buford Highway, NE, Mail-Stop K-67, Atlanta, GA, 30341, USA; 2 Division of Behavioral Surveillance, Office of Surveillance, Epidemiology and Laboratory Service, Centers for Disease Control and Prevention (CDC), 2500 Century Parkway, Atlanta, GA, 30345, USA

**Keywords:** Insufficient sleep, Chronic disease, Population-based study

## Abstract

**Background:**

Although evidence suggests that poor sleep is associated with chronic disease, little research has been conducted to assess the relationships between insufficient sleep, frequent mental distress (FMD ≥14 days during the past 30 days), obesity, and chronic disease including diabetes mellitus, coronary heart disease, stroke, high blood pressure, asthma, and arthritis.

**Methods:**

Data from 375,653 US adults aged ≥ 18 years in the 2009 Behavioral Risk Factor Surveillance System were used to assess the relationships between insufficient sleep and chronic disease. The relationships were further examined using a multivariate logistic regression model after controlling for age, sex, race/ethnicity, education, and potential mediators (FMD and obesity).

**Results:**

The overall prevalence of insufficient sleep during the past 30 days was 10.4% for all 30 days, 17.0% for 14–29 days, 42.0% for 1–13 days, and 30.6% for zero day. The positive relationships between insufficient sleep and each of the six chronic disease were significant (p < 0.0001) after adjustment for covariates and were modestly attenuated but not fully explained by FMD. The relationships between insufficient sleep and both diabetes and high blood pressure were also modestly attenuated but not fully explained by obesity.

**Conclusions:**

Assessment of sleep quantity and quality and additional efforts to encourage optimal sleep and sleep health should be considered in routine medical examinations. Ongoing research designed to test treatments for obesity, mental distress, or various chronic diseases should also consider assessing the impact of these treatments on sleep health.

## Background

Although sleep is a necessity, about 60 million Americans are affected by chronic sleep disorders and sleep problems that can impair physical well-being and cognitive functioning [1]. A growing body of evidence strongly suggests that self-reported sleep durations are correlates of diabetes, cardiovascular disease, obesity, depression and anxiety [2]. These include a cross-sectional study [3], prospective cohort studies [4-6], and an intervention study [7]. However, underlying mechanisms of this relationship are still widely discussed.

Recently, the Centers for Disease Control and Prevention (CDC) expanded the information collected on sleep health in the U.S. national surveillance systems based on recommendations from the Institute of Medicine [1]. Thus, the Behavioral Risk Factor Surveillance System (BRFSS), the largest telephone health survey in the world, began collecting data on perceived insufficient sleep, defined by the number of days the respondent felt that he/she did not get enough rest or sleep during the past 30 days [8]. Although polysomnography in a sleep clinic provides a more objective measure of sleep loss and sleep quality than a subjective measure of insufficient sleep, it is not feasible for large national surveillance systems. Furthermore, perceived insufficient sleep is similar to a sleep complaint measure provided in a primary care setting to indicate a concern or problem with sleep quality and quantity such as sleep disturbance and other symptoms of sleep disorders. Several reports on insufficient sleep in BRFSS to date have addressed the prevalence of insufficient sleep [9] and the association between perceived insufficient rest/sleep and cardiovascular disease, diabetes mellitus, obesity [10-12] and smoking habits [13]. In contrast to self-reported sleep duration, insufficient sleep is a less studied dimension of sleep experience which may provide additional important information for understanding the role of sleep in general health at the population level. As depression and obesity are associated with impaired sleep [10,14-16] and several health outcomes [17-22], the present study aims to examine the relationships between perceived insufficient sleep and other chronic diseases such as high blood pressure, arthritis, and asthma, and to assess whether the relationships between perceived insufficient sleep and chronic disease may be attenuated by frequent mental distress (FMD) and obesity.

## Methods

### Survey design

The BRFSS is a large annual, random-digital-dialed telephone survey conducted in all 50 states, the District of Columbia, and US territories. The BRFSS collects data on health-related behaviors that are linked to chronic disease and other conditions among the non-institutionalized US civilian population (aged ≥18 years) living in households with landline telephones. Trained interviewers administer standardized questionnaires to all survey participants. Although the response rate in the 2009 BRFSS varied among states (ranged from 37.9% to 66.9%, median = 52.5%) [23], BRFSS data have been verified as being of high quality and very reliable [24]. A study also indicated that bias was not associated with response rate in the BRFSS survey due to the study design [25]. Of 424,592 adults in the 50 states and the District of Columbia who responded to the 2009 BRFSS survey, 375,653 (88.5%) respondents were included in our analysis after we excluded pregnant women (n = 2,358), persons who reported missing value on days of insufficient sleep (n = 7,336), any of six chronic diseases (n = 10,347), or other variables of interest (n = 28,898).

A detailed description of the BRFSS survey design, data collection, and full-text questionnaires can be found at http://www.cdc.gov/brfss. The BRFSS study has been approved by Human Research Review Boards from state departments of health.

### Outcome of interest

The occurrence of six chronic diseases was based on affirmative responses to respondents’ being asked if they had ever been told by a doctor or other health professional that they had diabetes mellitus, coronary heart disease (CHD, a heart attack, angina pectoris, or coronary heart disease), stroke, high blood pressure, asthma, or arthritis. Persons who reported “don’t know/not sure” were defined as not having the condition. Analyses were repeated after those who chose ‘do not know/not sure’ on the six chronic diseases were excluded in order to assess the potential misclassification of these respondents due to a less rigorous classification. No significant difference in the results was observed. Those who reported having borderline diabetes or pre-diabetes or having diabetes only during pregnancy were defined as not having diabetes mellitus. Those who reported having borderline high blood pressure, being pre-hypertensive, or having high blood pressure only during pregnancy were defined as not having high blood pressure.

### Exposure variable

Perceived insufficient sleep was assessed by the question “During the past 30 days, for about how many days have you felt you did not get enough rest or sleep?” For these analyses, the number of days of insufficient sleep was categorized as 0 day, 1–13 days, 14–29 days, and 30 days.

### Assessment of covariates

The socio-demographic characteristics that were examined as covariates in the association between perceived insufficient sleep and these chronic diseases included sex, age in years (18–24, 25–34, 35–44, 45–64, and ≥65), race/ethnicity (non-Hispanic white, non-Hispanic black, Hispanic, and other non-Hispanic), and education (less than high school graduate, high school diploma or GED recipient, some college, and college graduate). The respondent was defined as having frequent mental distress (FMD) if he/she responded ≥14 days to the following question “Now thinking about your mental health, which includes stress, depression and problems with emotions, for how many days during the past 30 days was your mental health not good?” [26]. Assessment of obesity was based on the body mass index (BMI, kg/m^2^) calculated from self-reported height in inches and weight in pounds (underweight: BMI < 18.5 kg/m^2^; normal weight: BMI = 18.5-24.9 kg/m^2^; overweight: BMI = 25.0-29.9 kg/m^2^; obese: BMI ≥ 30 kg/m^2^) [27].

### Statistical analyses

First, we examined the distribution of selected characteristics in the study population and the prevalence of ≥14 days of insufficient sleep by selected characteristics. We also assessed the relationships of FMD and obesity to each of the chronic diseases with multivariate logistic regression analyses. Then separate univariate and multivariate logistic regression analyses were performed to examine the relationship of days of insufficient sleep with each of the six chronic diseases without and with adjustment for age, gender, race/ethnicity, and education. The magnitude and significance of effect was assessed using the adjusted odds ratios (ORs) and 95% confidence interval (CI). We also assessed whether FMD and/or obesity were potential mediators in the relationship between days of insufficient sleep and each chronic disease by measuring the percentage change in effect between a model with and without the specific proposed mediator = [(OR _model without the mediator_- OR _model with the mediator_)/ (OR _model without the mediator_ - 1)] * 100 [28,29]. To be conservative, 20% of the percentage change or more was presented here [29] only if the following three criteria were met for determining mediation: 1) there must be a significant relationship between insufficient sleep and chronic disease; 2) there must be a significant relationship between insufficient sleep and either FMD or obesity; 3) there must be a significant relationship between either FMD or obesity and chronic disease [30].

All analyses were conducted using SAS-callable SUDAAN to account for the complex sampling design [SUDAAN, version 10.0.1, Research Triangle Institute, NC]. The significance level was denoted at 0.01 due to large sample size.

## Results

Of 375,653 adult respondents in the current study population, about half were aged ≥45 years (51.4%) and women (49.7%), 70.8% were non-Hispanic white, and 62.2% had more than a high school diploma (Table 1). Among participants, 65.5% reported no days of mental distress and 10.6% reported having FMD, 27.5% were obese, 8.9% had diabetes, 6.1% had a history of coronary heart disease, 2.5% had stroke, 29.2% had high blood pressure, 13.4% reported a history of asthma, and 25.9% reported arthritis. At least one of the six chronic diseases was reported by 51.7% of survey respondents. Only 30.6% reported no days of insufficient sleep, while 42.0% reported 1–13 days, 17.0% reported 14–29 days, and 10.4% reported every day of insufficient rest or sleep in the past 30 days.

**Table 1:** Distribution of selected characteristics among adults aged ≥18 years, behavioral risk factor surveillance system, 2009

***Characteristic***	**n**^**1**^	**%**^**2**^	**(95% CI)**^**2**^
**Total**	375,653	100.0	
**Age, years**			
18-24	11,038	11.4	(11.1-11.7)
25-34	31,443	18.0	(17.7-18.3)
35-44	52,819	19.2	(18.9-19.4)
45-64	161,886	34.4	(34.1-34.7)
≥65	118,467	17.0	(16.8-17.2)
**Sex**			
Men	147,785	50.3	(50.0-50.7)
Women	227,868	49.7	(49.3-50.0)
**Race/Ethnicity**			
Non-Hispanic White	305,878	70.8	(70.4-71.1)
Non-Hispanic Black	28,539	9.9	( 9.7-10.2)
Hispanic	21,094	12.7	(12.4-13.0)
Non-Hispanic others	20,142	6.6	( 6.4- 6.8)
**Education Attainment**			
Less than a high school graduate	32,053	9.7	( 9.5-10.0)
High school graduate or GED	111,589	28.0	(27.7-28.4)
Some college	102,119	26.9	(26.6-27.2)
College graduate	129,892	35.3	(35.0-35.6)
**Mental Distress, days/last 30 days**			
0	258,295	65.5	(65.1-65.9)
1-13	78,401	23.9	(23.5-24.3)
**≥**14	38,957	10.6	(10.4-10.8)
**Body Mass Index, kg/m**^**2**^			
Underweight (<18.5)	5,843	1.7	(1.6-1.8)
Normal Weight (18.5-24.9)	125,397	34.5	(34.2-34.8)
Overweight (25.0-29.9)	137,753	36.3	(36.0-36.6)
Obese (≥30.0)	106,660	27.5	(27.2-27.8)
**Diabetes**	44,607	8.9	(8.7-9.1)
**Coronary Heart Disease**	33,735	6.1	( 6.0- 6.2)
**Stroke**	14,744	2.5	( 2.4- 2.6)
**High Blood Pressure**	145,719	29.2	(29.0-29.5)
**Asthma**	48,601	13.4	(13.2-13.7)
**Arthritis**	138,360	25.9	(25.6-26.1)
**Number of chronic diseases**			
None	138,802	48.3	(48.0-48.6)
1	114,143	28.7	(28.4-29.0)
2	74,356	14.6	(14.4-14.8)
3	34,004	6.0	( 5.9- 6.1)
≥4	14,348	2.4	( 2.3- 2.5)
**Insufficient Sleep, days/last 30 days**			
0	137,314	30.6	(30.3-30.9)
1-13	146,218	42.0	(41.7-42.4)
14-29	54,998	17.0	(16.8-17.2)
30	37,123	10.4	(10.2-10.6)

^1^Unweighted sample size.

^2^Percentages and 95% confidence interval (CI) were calculated using sampling weights.

Table 2 reveals the prevalence of ≥14 days of perceived insufficient sleep by selected demographics. Compared to their peers, respondents aged 25–44 years, women, non-Hispanic blacks, and respondents who reported having either FMD or obesity reported a higher percentage of frequent insufficient sleep (p < 0.001). In addition, college graduates were less likely to report frequent insufficient sleep than persons with other levels of education attainment (p < 0.01).

**Table 2:** The prevalence of frequent insufficient sleep by selected characteristics, BRFSS, 2009

**Characteristic**	**n**^**1**^	**Frequent insufficient sleep (≥14 days/the past 30 days), % (95% CI)**^**2**^
Total	375,653	27.4 (27.1-27.7)
**Age, years**		
18-24	11,038	29.3 (27.9-30.8)
25-34	31,443	35.6 (34.7-36.6)
35-44	52,819	32.6 (31.8-33.3)
45-64	161,886	26.1 (25.7-26.5)
≥65	118,467	14.1 (13.7-14.4)
**Sex**		
Men	147,785	24.9 (24.4-25.4)
Women	227,868	29.9 (29.5-30.3)
**Race/ethnicity**		
Non-Hispanic White	305,878	27.3 (26.9-27.6)
Non-Hispanic Black	28,539	29.4 (28.3-30.5)
Hispanic	21,094	26.5 (25.3-27.7)
Non-Hispanic others	20,142	27.3 (25.9-28.6)
**Education attainment**		
Less than a high school graduate	32,053	29.0 (27.8-30.1)
High school graduate or GED	111,589	27.9 (27.3-28.5)
Some college	102,119	29.7 (29.1-30.4)
College graduate	129,892	24.7 (24.3-25.2)
**Frequent mental distress (FMD, ≥14 days/30 days)**		
Yes	38,957	61.8 (60.7-62.8)
No	336,696	23.3 (23.0-23.6)
**Obesity (BMI ≥30 kg/m**^**2**^**)**		
Yes	106,660	32.0 (31.4-32.6)
No	268,993	25.6 (25.2-25.9)

^1^Unweighted sample size.

^2^Percentages and 95% CI were calculated using sampling weights.

Table 3 demonstrated that there are significant relationships for both FMD and obesity with each of the six chronic disease (p < 0.0001). Furthermore, the regression coefficients for the relationships of FMD were strong especially with CHD, stroke, and arthritis. The regression coefficients for the relationships of obesity with diabetes and high blood pressure were also greater than that with the other chronic diseases.

**Table 3:** Regression coefficients of chronic disease related to frequent mental distress and obesity, BRFSS, 2009

**Chronic Disease (outcome variables)**	**Frequent mental distress (FMD) Beta (SE)**^**1**^	**Obesity Beta (SE)**^**1**^
Diabetes	0.55 (0.03)^2^	1.17 (0.02)^2^
High Blood Pressure	0.54 (0.02)^2^	0.99 (0.02)^2^
Coronary Heart Disease	0.90 (0.04)^2^	0.41 (0.03)^2^
Stroke	0.93 (0.04)^2^	0.18 (0.04)^2^
Asthma	0.62 (0.03)^2^	0.42 (0.02)^2^
Arthritis	0.87 (0.02)^2^	0.61 (0.02)^2^

^1^The regression coefficients were obtained from separate multivariate logistic regression models that assessed the relationship of either frequent mental distress (FMD) or obesity to the chronic disease and included age, sex, race/ethnicity, and education as covariates.

^2^p < 0.0001.

The prevalence of each of the six chronic diseases and the adjusted odds ratio (OR) for the likelihood of having any of the chronic diseases by the four categories of insufficient sleep were obtained from multivariate logistic regression analyses (Table 4). Specifically, significant graded relationships were observed at each sleep level with high blood pressure, arthritis, and asthma. At insufficient sleep levels of both 14–29 days and ≥30 days, the likelihoods of having diabetes, CHD, and stroke was greater compared to persons with zero day. FMD moderately attenuated (at least 20-40%) but did not completely explain the significant relationships of insufficient sleep with the six chronic diseases (Model 2). Additionally, the likelihoods of having diabetes and high blood pressure associated with insufficient sleep were moderately attenuated (20-40%) but were not fully explained by obesity (Model 3).

**Table 4:** Odds ratios (OR) and 95% confidence intervals (CI) for the likelihoods of having chronic diseases associated with categories of insufficient sleep among adults, behavioral risk factor surveillance system (BRFSS), 2009

**Number of days insufficient sleep in past 30 days**	**Unadjusted prevalence**	**Model 1**^**1**^	**Model 2**^**2**^	**Model 3**^**3**^
	**% (95% CI)**	**Adjusted OR (95% CI)**	**Adjusted OR (95% CI)**	**Adjusted OR (95% CI)**
**Diabetes**				
0	6.4 ( 6.1- 6.7)	1.00 (referent)	1.00 (referent)	1.00 (referent)
1-13	5.8 ( 5.5- 6.0)	0.96 (0.91-1.01)	0.95 (0.90-1.00)	0.93 (0.88-0.98)
14-29	8.0 ( 7.5- 8.5)	1.35 (1.26-1.45)	1.24 (1.16-1.34)^4^	1.25 (1.16-1.34)^4^
30	10.2 ( 9.6-10.7)	1.65 (1.54-1.76)	1.46 (1.36-1.56)^4^	1.48 (1.38-1.59)^4^
**Coronary Heart Disease**				
0	3.2 ( 2.9- 3.4)	1.00 (referent)	1.00 (referent)	1.00 (referent)
1-13	2.8 ( 2.6- 3.0)	0.96 (0.91-1.01)	0.95 (0.90-1.00)	0.95 (0.89-1.00)
14-29	4.5 ( 4.2- 4.8)	1.58 (1.47-1.70)	1.38 (1.28-1.49)^4^	1.54 (1.43-1.65)
30	6.6 ( 6.1- 7.1)	2.26 (2.09-2.45)	1.86 (1.71-2.02)^4^	2.18 (2.01-2.36)
**Stroke**				
0	1.4 ( 1.2- 1.5)	1.00 (referent)	1.00 (referent)	1.00 (referent)
1-13	1.2 ( 1.1- 1.3)	0.91 (0.84-0.98)	0.89 (0.82-0.97)	0.90 (0.83-0.98)
14-29	1.8 ( 1.6- 1.9)	1.31 (1.19-1.45)	1.11 (1.01-1.23)^5^	1.30 (1.18-1.44)
30	3.0 ( 2.7- 3.3)	2.11 (1.91-2.33)	1.68 (1.50-1.87)^4^	2.08 (1.88-2.30)
**High Blood Pressure**				
0	24.1(23.6-24.7)	1.00 (referent)	1.00 (referent)	1.00 (referent)
1-13	24.8 (24.3-25.3)	1.09 (1.05-1.13)	1.08 (1.04-1.12)	1.08 (1.04-1.12)
14-29	29.7 (28.9-30.5)	1.40 (1.34-1.47)	1.30 (1.23-1.36)^4^	1.31 (1.25-1.38)^4^
30	33.5 (32.5-34.5)	1.59 (1.51-1.68)	1.41 (1.34-1.49)^4^	1.46 (1.38-1.54)^4^
**Asthma**				
0	10.2 ( 9.8-10.6)	1.00 (referent)	1.00 (referent)	1.00 (referent)
1-13	12.5 (12.1-12.9)	1.24 (1.17-1.31)	1.23 (1.16-1.30)	1.23 (1.16-1.30)
14-29	17.1 (16.5-17.8)	1.76 (1.65-1.88)	1.63 (1.53-1.74)	1.70 (1.60-1.82)
30	19.7 (18.8-20.5)	2.06 (1.92-2.21)	1.82 (1.70-1.96)^4^	1.99 (1.85-2.13)
**Arthritis**				
0	16.4 (16.0-16.8)	1.00 (referent)	1.00 (referent)	1.00 (referent)
1-13	20.0 (19.6-20.4)	1.26 (1.22-1.30)	1.25 (1.21-1.29)	1.25 (1.20-1.29)
14-29	29.5 (28.8-30.3)	2.06 (1.97-2.15)	1.85 (1.76-1.93)	1.98 (1.90-2.08)
30	35.5 (34.5-36.5)	2.65 (2.51-2.78)	2.23 (2.12-2.35)^4^	2.52 (2.39-2.66)

^1^Model 1: adjusted odds ratios (OR) and 95% confidence interval (CI) were obtained from separate multivariate logistic models that included days of insufficient sleep and age, sex, race/ethnicity, and education as covariates.

^2^Model 2: includes the covariates in model 1 plus frequent mental distress (FMD).

^3^Model 3: includes the covariates in model 1 plus categorical body mass index (BMI).

^4^20-40% reduction in the effect due to the addition of a mediator.

^5^ >60% reduction in the effect due to the addition of a mediator.

## Discussion

Our study demonstrates two important findings. First, to our knowledge, it is the first report to reveal significant relationships between insufficient sleep and high blood pressure, asthma, and arthritis. In addition, significant relationships between insufficient sleep and obesity, diabetes, CHD, and stroke are consistent with recent reports [11,12,31]. Second, these significant associations were still present after adjustment for age, sex, race/ethnicity, education, FMD, and obesity. FMD and obesity were moderate mediators but did not fully explain the relationships of insufficient sleep to these chronic diseases. Therefore, insufficient sleep appears to be an important correlate of the leading chronic diseases.

In the BRFSS insufficient sleep is a newly developed sleep indicator and is not accompanied by reports of hours of sleep per night in the full study population. As an ad hoc analysis, we analyzed data from 74,944 respondents who had information on both insufficient sleep and sleep duration in 12 states in 2009. Our results indicate that the prevalence of reporting ≥14 days of perceived insufficient sleep was greater among those reporting <7 hours sleep (51.0%) in contrast to those reporting 7–9 hours (12.1%) or ≥10 hours sleep (16.1%) in a 24-hour period. Furthermore, the percentage of those reporting an optimal sleep (7–9 hours) also differed by number of days of perceived insufficient sleep (78.2% of 0 day, 67.4% of 1–13 days, 32.4% of 14–29 days, and 19.7% of all 30 days). These results are consistent with the findings from a previous study in Finland [32] and suggest that insufficient sleep is strongly related to sleep duration but they definitely are not redundant because they share only partially same information. Additionally, insufficient sleep may partly reflect some sleep disorders such as obstructive sleep apnea, which is also associated with chronic diseases [33-36] but we could not assess that relationship due to lack of data. Considerable data suggest that chronic sleep loss can result in insulin resistance and changes in appetite-regulating hormones, such as increased ghrelin and decreased leptin. The latter conditions further develop into chronic metabolic impairments such as obesity, and may subsequently lead to the development of diabetes, hypertension, heart disease, stroke, and even mortality [37-39].

Experimental evidence suggested that sleep loss was associated with exacerbation of arthritis through the increase of the sensitivity of pain among persons with rheumatoid arthritis [40,41]. However, further study is needed to understand the directionality of the association between sleep loss and arthritis. In addition, a few studies indicated that poor sleep may affect lung function or lower the quality of life to exacerbate symptoms of asthma among youth [42,43] or worsen the bronchoconstriction among persons with nocturnal asthma [44]. The evidence may shed light on the mechanism of sleep loss associated with asthma.

Depression and anxiety often co-exist with sleep loss and many chronic diseases [45,46]. Consistent with previous data from prospective studies, our study demonstrated a highly significant relationship between insufficient sleep and FMD, an indicator of psychological distress, and between FMD and chronic disease [10,47]. Furthermore, our results suggested that FMD partially mediated the relationships between frequent insufficient sleep and chronic diseases although prospective studies are needed to clarify the pathway.

We assessed obesity as a potential mediator in the relationship between insufficient sleep and chronic disease because sleep loss is associated with increased risk for obesity and obesity is a robust risk factor for diabetes, hypertension, and cardiovascular diseases [3,6,11]. Our findings that obesity might partially mediate the relationships between frequent insufficient sleep and diabetes and high blood pressure were consistent with the results of studies assessing sleep duration [3,4].

Strengths of this study include the large sample size in this population-based study (N = 375,653). Furthermore, this study represents the adult population in each of the 50 states. However, our study is subject to several limitations. First, due to the cross-sectional nature of the study, it is not possible to confirm whether there is a causal relationship between insufficient sleep and chronic disease. Nevertheless, such a causal link is biologically plausible because sleep loss may lead to metabolic abnormalities and weight gain through the regulation of hypothalamic-pituitary-adrenal axis [47,48]. In addition, it is also very difficult to determine whether FMD and chronic conditions result in insufficient sleep or whether the direction of effect is reversed. Second, perceived insufficient sleep is a subjective measure of sleep health and has not been validated with polysomnography or other objective measures of sleep loss. Therefore, our results may have bias due to the misclassifications of exposure variable. Third, a significantly higher percentage of short sleep duration (average ≤6 hours per 24–hour period) was recently reported among workers with regular night shifts than among those with regular daytime shifts [49] supporting the role of circadian rhythm disruption in short sleeper and the possible association with chronic diseases [1]. Therefore, shift work status may affect our results. However, we are unable to assess the effect of shift work status or circadian rhythm disruption on the relationship of chronic disease with insufficient sleep due to lack of data in BRFSS. Fourth, although we excluded those respondents who had missing values from the analyses, this is likely to bias our results toward the null if those non-respondents were included in our analyses [28]. The results from a repeated analysis also confirm this assumption after including those non-respondents (data not shown). Additionally, self-reports may lead to underreporting of chronic diseases and obesity [50,51], which could result in a much stronger relationship of insufficient sleep with the outcomes in this study. Finally, sample selection bias due to the low response rate is also possible because persons with severely impaired physical or mental health might not complete the BRFSS. Institutionalized persons and persons residing in households without landline telephones were also not included in the survey. However, the effect of potential systematic bias might be limited as a significant relationship between insufficient sleep and chronic disease revealed in our study was consistent with the results from prior research measuring sleep duration [2-6].

## Conclusions

A positive relationship was observed between frequent insufficient sleep and six chronic diseases. These significant relationships were moderately attenuated by FMD and by obesity. Poor sleep can usually be ameliorated by improved sleep habits, weight loss, continuous positive airway pressure, oral devices, oral-nasal surgery, or pharmacological interventions [52,53]. Therefore, assessment of sleep quantity and quality and additional efforts to encourage optimal sleep and sleep health should be considered in routine medical examinations. Ongoing research designed to test treatments for obesity, mental distress, or various chronic diseases should also consider assessing the impact of these treatments on sleep health.

## Abbreviations

BRFSS: Behavioral risk factor surveillance system; CDC: Centers for disease control and prevention; FMD: Frequent mental distress; CHD: Coronary heart disease; BMI: Body mass index.

## Competing interests

The authors declare that they have no competing interests.

## Authors’ contributions

YL and JBC developed the conceptual model. YL performed data analysis and drafted the manuscript under the supervision of JBC. JBC, AGW, GSP, DPC, THS, LRM, and LP contributed to the interpretation of the results and discussion. All authors read and approved the final manuscript.

## Pre-publication history

The pre-publication history for this paper can be accessed here:

http://www.biomedcentral.com/1471-2458/13/84/prepub
